# DNA Damage in Chronic Heart Failure: Consequences Beyond those in the Heart

**DOI:** 10.36660/abc.20190884

**Published:** 2020-02

**Authors:** Camila Renata Corrêa, Jéssica Leite Garcia

**Affiliations:** 1 Departamento de Patologia da Faculdade de Medicina da Universidade Estadual Paulista Júlio de Mesquita Filho (Unesp), Botucatu, SP - Brazil

**Keywords:** DNA/genetics, Heart Failure/physiopathology, Myocardial Infarction, Tissue Distribution, Oxidative Stress, Rats Inbread

Chronic heart failure (CHF) affects approximately 1% to 2% of the population of developed countries and its prevalence increases approximately 1% in individuals aged 55 to 64 years and up to 17.4% in individuals aged 85 years and over.^1,2^ CHF is a complex disease with multiple causes and myocardial infarction (MI) is the most common cause. It is characterized by structural or functional cardiac alterations, affecting the ventilatory mechanics that impairs the oxygen uptake and supply to the systems, inducing oxidative stress.^2^

Oxidative stress, also currently called redox imbalance,^3^ is known to be associated with the development of several pathologies, either as a trigger or consequence. The biological system of redox reactions may break out of its equilibrium status when the formation of oxidizing species overcomes the antioxidant defense. This scenario favors the oxidation of biomolecules (lipids, proteins, DNA) resulting in their structural and functional damage, that is, contributing to significant pathological outcomes.^4^

The research published in this issue of the *Arquivos Brasileiros de Cardiologia* aimed at evaluating DNA damage in different tissues, such as the left ventricle, lungs and skeletal muscles (diaphragm, gastrocnemius and soleus) in rats submitted to MI to induce CHF.^5^ The authors' interest in assessing the influence of this pathology on other tissues is very relevant, as it shows the consequences of this condition on organs other than the heart. The indicator evaluated in this study was the DNA, a biomolecule vulnerable to several agents that can cause damage.^6^ Under normal conditions, approximately 99% of DNA damage can be repaired, but approximately 1% can remain in the cell genome.^7^ Unrepaired DNA damage can result in loss of genetic information, or interference with transcription and replication, therefore being deleterious to the organism.^6^ Another important aspect is that DNA damage may induce mutations^8,9^ that may be linked to several diseases, including cancer.^10^ Thus, DNA damage detection is an important element in studies related to disease development.

The study shows that DNA damage was remarkably higher in all organs evaluated in the CHF group, probably justified by the hyperfusion at these sites, which generated a prooxidative state that is toxic to this biomolecule. Although this study analyzed global DNA damage, which may be generated for reasons other than oxidative ones, human studies have already shown the presence of O8-OHdG, a product generated by purine oxidation, in the plasma of patients with CHF, confirming that this disease causes oxidative DNA damage.

Therefore, the results of the present study confirm that there are consequences in different organs resulting from CHF and that investigations should be carried out to minimize future complications.
